# Therapy-resistant breast cancer in focus: Clinically relevant mitigation by flavonoids targeting cancer stem cells

**DOI:** 10.3389/fphar.2023.1160068

**Published:** 2023-04-06

**Authors:** Alena Mazurakova, Lenka Koklesova, Desanka Vybohova, Marek Samec, Erik Kudela, Kamil Biringer, Miroslava Šudomová, Sherif T. S. Hassan, Martin Kello, Dietrich Büsselberg, Olga Golubnitschaja, Peter Kubatka

**Affiliations:** ^1^ Department of Anatomy, Jessenius Faculty of Medicine, Comenius University in Bratislava, Martin, Slovakia; ^2^ Clinic of Obstetrics and Gynecology, Jessenius Faculty of Medicine, Comenius University in Bratislava, Martin, Slovakia; ^3^ Department of Pathological Physiology, Jessenius Faculty of Medicine, Comenius University in Bratislava, Martin, Slovakia; ^4^ Museum of Literature in Moravia, Rajhrad, Czechia; ^5^ Department of Applied Ecology, Faculty of Environmental Sciences, Czech University of Life Sciences Prague, Prague, Czechia; ^6^ Department of Pharmacology, Faculty of Medicine, Pavol Jozef Safarik University, Kosice, Slovakia; ^7^ Department of Physiology and Biophysics, Weill Cornell Medicine in Qatar, Qatar Foundation, Doha, Qatar; ^8^ Predictive, Preventive and Personalised (3P) Medicine, Department of Radiation Oncology, University Hospital Bonn, Rheinische Friedrich-Wilhelms-Universität Bonn, Bonn, Germany; ^9^ Department of Medical Biology, Jessenius Faculty of Medicine, Comenius University in Bratislava, Martin, Slovakia

**Keywords:** breast cancer, resistance, non-responsiveness, cancer stem cells, predictive, preventive and personalised medicine, secondary and tertiary care

## Abstract

Significant limitations of the reactive medical approach in breast cancer management are clearly reflected by alarming statistics recorded worldwide. According to the WHO updates, breast malignancies become the leading cancer type. Further, the portion of premenopausal breast cancer cases is permanently increasing and demonstrates particularly aggressive patterns and poor outcomes exemplified by young patients with triple-negative breast cancer that lacks targeted therapy. Accumulating studies suggest the crucial role of stem cells in tumour biology, high metastatic activity, and therapy resistance of aggressive breast cancer. Therefore, targeting breast cancer stem cells is a promising treatment approach in secondary and tertiary breast cancer care. To this end, naturally occurring substances demonstrate high potential to target cancer stem cells which, however, require in-depth analysis to identify effective anti-cancer agents for cost-effective breast cancer management. The current article highlights the properties of flavonoids particularly relevant for targeting breast cancer stem cells to mitigate therapy resistance. The proposed approach is conformed with the principles of 3P medicine by applying predictive diagnostics, patient stratification and treatments tailored to the individualised patient profile. Expected impacts are very high, namely, to overcome limitations of reactive medical services improving individual outcomes and the healthcare economy in breast cancer management. Relevant clinical applications are exemplified in the paper.

## 1 Introduction

Breast cancer is at the forefront of incidence and mortality in women (Arnold et al., 2022). Based on the most recent GLOBOCAN statistics, in 2020, breast cancer was the most diagnosed cancer represented by 11.7% of all cancer cases and 24.5% of female cancer cases. Also, in the same year, breast cancer, the leading cause of cancer death, accounted for 15.5% of mortality in females (Sung et al., 2021). Moreover, Arnold et al. (2022) indicate the growth of newly diagnosed breast cancer cases by over 40% for incidence and by more than 50% for mortality by 2040. Current trends also highlight the rapid growth of the premenopausal cohort of BC patients. Thus, the ratio shift between post-menopausal and pre-menopausal breast cancer results in a younger breast-cancer profile compared with the previous century. Moreover, breast cancer in young women is more frequently asymptomatic, which is associated with difficulties in early diagnosis and treatment (Kudela et al., 2019). Alarming statistics are attributed to aggressive breast cancer subtypes, such as triple-negative breast cancer (TNBC) (Vaz-Luis et al., 2017; Howard and Olopade, 2021), which is more typical for premenopausal women (Kudela et al., 2019). Breast cancer, therefore, represents a severe healthcare and socio-economic problem (Mazurakova et al., 2022a).

Despite the progress in cancer management and early detection, cancer still remains a serious global problem. Cancer stem cells (CSCs) represent a sub-population of cancer cells that are characterized by self-renewal, differentiation, stemness, clonal expansion, promotion of cancer recurrence, and resistance to standard therapeutics (Wang et al., 2018; Tsai et al., 2021). The obstacle of the non-responsiveness or resistance of breast cancer to standard therapeutic options also requires in-depth analyses to identify effective anti-cancer agents applicable for adequate management of breast cancer, including the use of pleiotropic properties of phytochemicals (Mazurakova et al., 2022a). Current oncological research highlights the potential utilization of naturally occurring substances in the prevention and treatment of breast cancer (Kubatka et al., 2016; Kapinova et al., 2017; Kubatka et al., 2017; Abotaleb et al., 2018; Kapinova et al., 2019; Liskova et al., 2019; Kubatka et al., 2020a; Liskova et al., 2020a; Kubatka et al., 2020b; Liskova et al., 2020b). Flavonoids are plant secondary metabolites commonly found in vegetables, fruit, grains, or medicinal plants. Flavonoids exert potent anticancer capacity and pleiotropic effects targeting each of the multistep processes of carcinogenesis, including cancer initiation, promotion, and progression into metastatic diseases (Abotaleb et al., 2018; Jasek et al., 2019; Liskova et al., 2020a; Liskova et al., 2020b; Liskova et al., 2020c; Koklesova et al., 2021; Kubatka et al., 2021; Liskova et al., 2021; Samec et al., 2021). Therefore, flavonoids represents a promising source of phytochemicals with a potential to target CSCs.

Patient-beneficial and cost-effective management of breast cancer require the application of the principles of predictive, preventive, and personalized medicine (3PM), including advanced screening programs and targeted individualized approach in primary, secondary, and tertiary care (Bubnov et al., 2017; Fröhlich et al., 2018; Goldstein et al., 2020; Golubnitschaja et al., 2021). Here, we highlight the crucial benefits of flavonoids targeting breast cancer cells to combat the resistance of breast cancer in the framework of mainly secondary and tertiary care to improve individual outcomes and the cost-effectiveness of breast cancer management.

## 2 Breast cancer stem cells (BCSCs)

The stem cell theory describes the subpopulation of cancer cells entitled cancer stem cells (CSCs), also known as tumour-initiating cells. In the scope of breast cancer, CSCs can be termed breast cancer stem cells (BCSCs). BCSCs are characterized by a capability of self-renewal, plasticity, clonogenicity, and drug resistance (Liu et al., 2016), and by the presence of specific CSCs markers, including CD24, CD44, CD133, or ALDH1, among others (Ercan et al., 2011; Galoczova et al., 2018; Yousefnia et al., 2019). CSCs frequently overexpress ATB-binding cassette (ABC) efflux proteins that are responsible for the efflux of the drug outside the cell and thus contribute to the resistance to anti-cancer therapeutics. ABC transporters frequently overexpressed in BCSCs include multidrug-resistance-associated protein 1 (ABCC1, MRP1) and ATP-binding cassette superfamily G member 2 (ABCG2), also known as breast cancer resistance protein (BCRP) (Koh et al., 2019; Sridharan et al., 2019). Another molecule of the ATP-dependent proton pump family essential for the resistance of cancer cells is P-glycoprotein (P-gp), the product of *MDR1 (ABCB1)* gene (Nandi et al., 2022a).

The identification and isolation of CSCs from primary tumours or cancer cell lines can be performed *via* several methods including 1) detection of CSCs by side populations phenotypes based on high ABC transporter expression *via* hoechst 33,342 exclusion, 2) the enrichment of putative CSCs by sphere-formation in serum-free medium (based on the ability to form spheres in suspensions), 3) CSCs sorting by flow cytometry according to CSCs markers, and 4) isolation of CSC *via* measuring the activity of ALDH (Yousefnia et al., 2019; Zhang et al., 2020). Indeed, the identification of BCSCs phenotypes is based mainly on CD44, CD24, and ALDH1 while BCSCs with CD44^+^/CD24^−^ and ALDH1^+^ phenotypes are considered as most aggressive and thus frequently associated with resistance to treatments (Zhang et al., 2020). Another method of investigating the existence of cancer cells capable of self-renewal is performed *via* transplantation of cells characterized by the presence of specific cell surface markers into NOD/SCID mice to observe the potential to give rise to new tumours (Ercan et al., 2011).

### 2.1 BCSCs: Plasticity between mesenchymal-like and epithelial-like states

Epithelial-to-mesenchymal transition (EMT) is a process by which cancer cells convert from epithelial into mesenchymal phenotype and gain migratory and invasive properties (Liskova et al., 2020c). As stated by Nallanthighal et al. (2017), EMT represents a key process of CSCs generation and maintenance of their characteristics associated also with metastasis as well as therapy resistance (Luo et al., 2015). Radio resistant MDA-MB-231 cells established by Ko et al. (2020) show promoted aggressiveness, CSCs features, and EMT. Thus, EMT-induction can also be considered a mechanism of resistance to radiotherapy or chemotherapy, as a typical characteristic of BCSCs, and both represent a target of strategies to overcome therapeutic resistance. CSCs show genetic and phenotypic heterogeneity but also a capacity to maintain plasticity to transition between mesenchymal-like and epithelial-like states regulated by tumour microenvironment (Luo et al., 2015). Mesenchymal-like BCSCs represent CD44^+^/CD24^−/low^ populations that have EMT signature (low E-cadherin, high vimentin), tend to be quiescent (Luo et al., 2015; Kamble et al., 2021), and are localized at tumour-invasive edge adjacent to tumour stroma (Kotiyal and Bhattacharya, 2014). On the contrary, epithelial-like BCSCs characterized as ALDH^+^ populations possess signature of high E-cadherin and low vimentin, tend to be more proliferative (Luo et al., 2015; Kamble et al., 2021), and are located more centrally (Kotiyal and Bhattacharya, 2014). Despite differences between CSCs cells expressing either CD44^+^/CD24^−/low^ or ALDH^+^, both populations are characterized by stemness, ability to re-create tumours *in vivo*, and a similar pattern of gene expression across molecular subtypes of breast cancer (Zhang et al., 2020). Moreover, the small population of BCSCs that express both markers (CD44^+^/CD24^−/low^ and ALDH^+^) possess the greatest tumorigenic and metastatic capacity (Kotiyal and Bhattacharya, 2014; Zhang et al., 2020). Specific molecular breast cancer subtypes are characterized by different BCSCs populations. Basal-like tumours generally contain more CD44^+^/CD24^−/low^ and ALDH1^+^ than luminal breast cancer subtypes. Specifically, luminal breast cancer tumours are enriched in CD44^−/low^/CD24^+^ cell population, basal/epithelial in CD44^+^/CD24^+^ cell populations, and basal/mesenchymal in CD44^+^/CD24^−/low^ cell populations (Zhang et al., 2020). TNBC is a basal-like tumour representing the most aggressive molecular subtype of breast cancer that is characterized by the highest content of BCSCs with CD44^+^/CD24^−/low^ and ALDH1^+^ and is frequently responsible for therapeutic resistance (Zhang et al., 2020).

### 2.2 Characteristics of BCSCs related to therapy resistance

#### 2.2.1 Mammosphere formation

BCSCs tend to form spheroids, non-adherent spherical clusters of cells, also known as mammospheres (Liu et al., 2016). Mammospheres are considered a pre-cancerous state and an indicator of stem-ness (Bhandary et al., 2021; Patel et al., 2021), specifically self-renewal abilities and increased tumorigenicity and metastatic potential (Truong et al., 2021). Cells from mammospheres can be serially passaged and keep their multipotency (Ercan et al., 2011). Mammosphere culture of breast cancer cells is an effective way to enrich CSCs showing high tumorigenicity and chemoresistance (Wu et al., 2015). Stem-ness properties are analysed *via in vitro* assay in which BCSCs are enriched in suspension culture as “mammospheres” that imitate *in vivo* tumours (Wu et al., 2015; Manupati et al., 2017).

Interestingly, Yousefnia et al. (2019) performed a study using three different breast cancer cell lines with different molecular phenotypes to enrich BCSCs. For example, treatment with docetaxel resulted in significant resistance in MDA-MB-213-derived and SKBR3-derived mammospheres. Also, MCF-7-derived mammospheres showed more resistance to docetaxel when compared with parental MCF-7 cells. Accordingly, cell lines with different molecular properties possess different mammosphere formation and CSC characteristics. Although all tested cell lines showed increased mammosphere formation efficiency, MCF-7 cell line exerted the highest mammosphere formation, which may be affected by E-cadherin expression. Also, mammospheres resistant to letrozole showed increased stemness and invasive markers, while letrozole resistance is frequently associated with CSCs (Patel et al., 2021).

#### 2.2.2 Clonogenicity

High clonogenicity is a fundamental characteristic of BCSCs (Liu et al., 2016; Rajendran and Jain, 2018). Therefore, colony forming or clonogenic assay can be considered a measure of CSCs stemness (Esquer et al., 2020), which is performed for *in vitro* evaluation of the capability of a single cell to grow into a colony *via* clonal expansion (Rajendran and Jain, 2018). The tumour formation at the secondary site reflects the stemness potential of cancer cells, which can be tracked *via* detecting the colony-forming potential (Sharma et al., 2022). The exposure of tamoxifen in MCF-7 cells resistant to tamoxifen (MCF7-TR) resulted in an increased number of CSCs as observed by the increased number of ALDH+ cells accompanied by increased colony formation, a higher number of mammospheres, and alterations in epigenetic and multidrug resistance (MDR) stem cell marker genes (Kalyanaraman et al., 2020). In addition, radioresistant breast cancer cells (RT-R-MDA-MB-231) showed increased colony formation and expression of CD44 and other markers of CSCs such as Notch, ALDH1, or OCT3/4 (Ko et al., 2018).

#### 2.2.3 Side population

Side population (SP) cells represent a sub-population of cancer cells that contain a higher proportion of CSCs or exhibit CSC-like properties, overexpress ABC transporters or other CSC markers, including Notch1, and contribute to the resistance of breast cancer cells (Britton et al., 2011; Wang et al., 2015; Liu et al., 2016). As reviewed by Britton et al. (2011), SP cells are frequently radio-resistant and chemo-resistant. Indeed, the utilization of standard chemotherapeutics frequently result in SP enrichment, thus showing an inability to target these cells (Liu et al., 2016).

#### 2.2.4 Overactivated Nrf2

The maintenance of low ROS levels represents an essential characteristic of CSCs resulting from elevated expression of ROS-scavenging molecules and antioxidant enzymes favouring survival and resistance (Wu et al., 2015). The transcriptional factor nuclear factor erythroid 2-related factor 2 (Nrf2) is an important regulator of intracellular antioxidant responses *via* the regulation of transcription of genes localised in the antioxidant response element (ARE) (Kamble et al., 2021). Cancer cells are associated with a higher level of endogenous ROS when compared with normal cells. However, CSCs show high antioxidant capacity keeping ROS at lower levels (through upregulated ROS-scavenging systems such as Nrf2-mediated signalling), which promotes the stemness, survival, and drug resistance of CSC (Woo et al., 2017; Tsai et al., 2021). Above all, CSCs are characterized by constitutive activation of Nrf2 (Woo et al., 2017), which is associated with resistance to radiotherapy and chemotherapy (Kamble et al., 2021). Qin et al. (2021) describe Nrf2 upregulation and its nuclear translocation in a ROS-dependent manner as a result of exposure of TNBC cells to ionizing radiation. Thus, ROS induced by ionizing radiation can activate Nrf2, which counteracts irradiation’s killing effect. However, pharmacological inhibition of Nrf2 activation induced by ionizing radiation sensitized TNBC CSCs to irradiation. Moreover, Nrf2 knockdown suppressed the ALDH^+^ population, stem cell markers, and reduced radio-resistance by decreasing clonogenicity in immunocompromised mice (Kamble et al., 2021). Studies show elevated Nrf2 in chemo-resistant CSC-enriched breast tumours. Nrf2-associated antioxidant genes include, for example, heme oxygenase 1 (HO-1) (Kamble et al., 2021). Nrf2-silenced mammospheres resulted in increased cell death and reduced sphere growth associated with the repression of Nrf2 target genes. Specifically, attenuated efflux transporters (such as BCRP) and increased doxorubicin accumulation were observed in Nrf2 knockdown mammospheres when compared with control mammospheres (Ryoo et al., 2015).

#### 2.2.5 NANOG, SOX2, OCT4

CSCs are also defined by aberrant expression or overactivation of specific molecules (such as transcription factors NANOG, SOX2, OCT4) that promote stemness and therapeutic resistance (Nandi et al., 2022a). Transcription factor SOX2 is crucial for embryonic development; however, SOX2 tends to be overexpressed in CSCs and to contribute to therapeutic resistance (Novak et al., 2020). Cancer cells with high SOX2 show CSCs features and EMT phenotype (Mauro-Lizcano et al., 2022). NANOG-CD44-MDR1 network maintains stemness and chemo-resistance. NANOG regulates SOX2 to maintain the pluripotency of breast cancer cells. Moreover, the upregulation of SOX2 and OCT4 induced by NANOG depends on upregulated CD44-MDR1 network (Nandi et al., 2022a). The binding of hyaluronic acid to CD44 induces the activation of NANOG and associated STAT-3-mediated *MDR1* gene expression. Also, the binding of hyaluronic acid with CD44 supports the association between ankyrin and P-gp and multidrug flux (Bourguignon et al., 2008). Pham et al. (2011) stated that the knockdown of CD44 in CD44^+^CD24^−^ cells isolated from primary cultures of malignant breast tumours resulted in the differentiation of BCSCs into non-BCSCs cells with decreased tumorigenic-potential and altered expression of some stem-cell related genes. Moreover, CD44 is associated with EMT and therapeutic resistance as characteristics of CSCs. As stated above, BCSCs are associated with developing resistance associated with the regulation of NANOG, SOX2, OCT4, CD44, and MDR1 (Nandi et al., 2022a). For example, MDA-MB-231 and MCF-7/tamoxifen-resistant cells showed overexpression of NANOG, OCT3/4, and SOX2 (Arif et al., 2015). Moreover, the expression of OCT4 and NANOG can also be promoted by Nrf2 (Tsai et al., 2021). Above all, differentiated breast cancer cells can be reprogrammed into BCSCs through the re-expression of stem-ness genes (*OCT4*, *SOX2*, *NANOG*, *KLF4*) induced by radiation (Lagadec et al., 2012; Wang et al., 2014).

**FIGURE 1:**
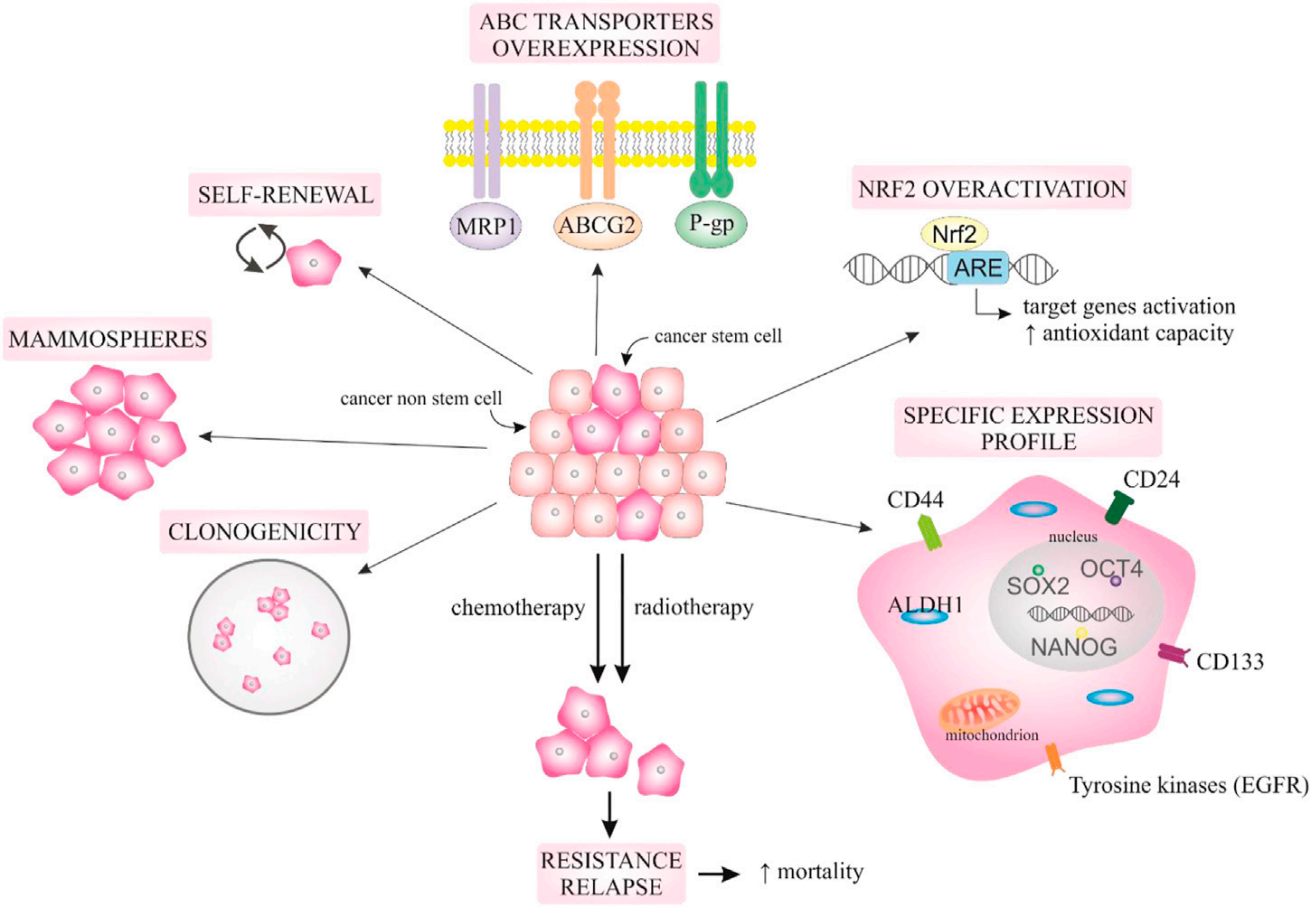
Selected BCSCs characteristics. Explanatory notes: BCSCs represent a sub-population of cancer cells that are defined by multiple characteristics including self-renewal capability, mammosphere formation, and clonogenicity. BCSCs are associated with aberrant expression of specific signalling molecules or aberrant activation of cell signalling pathways, including overexpression of ABC transporters (MRP1, ABCG2, P-gp responsible for the efflux of the drug outside the cell, overactivation of Nrf2 that increases antioxidant defence of cells, and overexpression of other markers such as CD44, CD24, EpCAM, tyrosine kinases such as EGFR, or expression transcription factors that contribute to the aggressive and resistant phenotype of BCSCs such as NANOG, SOX2, or OCT4) resulting in the regulation of target genes responsible for increased aggressiveness and therapeutic resistance that result in ineffective treatment and increased mortality of breast cancer patients.

Figure 1 provides an illustration of selected characteristics of BCSCs that contribute to their stem-ness and associated therapy resistance.

#### 2.2.6 Other BCSCs characteristics

Kruppel-like factor 4 (KLF4) is a product of an oncogene responsible for BCSCs maintenance, migration, and invasion. KLF4 is overexpressed in most breast cancers and is highly expressed in CSCs-enriched populations *in vivo* and breast cancer cell lines. Besides, KLF4 knockdown in breast cancer cells decresed the proportion of stem cells (Yu et al., 2011). Also, the abnormal expression of two main effectors of the Hippo pathway, YAP and TAZ (that mainly interacts with TEADs), are closely associated with tumorigenic processes, including the sustain of CSCs properties of self-renewal, tumour-initiating capabilities, and chemotherapy resistance particularly in TNBC. TNBC cell lines MDA-MB-231 and MDA-MB-436 frequently overexpress TAZ and are characterized by the presence of CD44^+^/CD24^−^ cells (Li et al., 2018a). Furthermore, breast cancer cells with CD44^+^/CD24^−/low^ phenotype (described as BCSCs) highly expressing Y-box binding protein 1 (YB-1) and P-gp are resistant to chemotherapeutics such as docetaxel, paclitaxel, or vincristine. YB-1 acts as a transcription factor in the nucleus and regulates translation in the cytoplasm; however, there is an evidence supporting the role of YB-1 in tumorigenesis, especially in the P-gp overexpression and CSCs enhancement (Li et al., 2018b). The chemokine receptor CXCR4 that is expressed in BCSCs is a crucial chemokine receptor that contributes to breast cancer metastasis, particularly of mesenchymal BCSCs. Therefore, CXCR4 also represents a target for BCSCs eradication (Sridharan et al., 2019). Other markers of CSCs include MUC1, a transmembrane glycoprotein that binds to the epidermal growth factor receptor (EGFR) family, and EpCAM that correlates with poor prognosis in numerous cancer patient types, including breast cancer (Wang et al., 2018). Moreover, Cripto-1 is a protein essential for human development in the early phases of gastrulation and is also associated with healing processes. However, Cripto-1 is upregulated in most cancer types and promotes cancer progression, metastasis, and CSCs survival (Arnouk et al., 2021). Besides, as reviewed by Tsai et al. (2021), Cripto-1 promoted EMT in mouse mammary tumours, which can be associated with EMT gene expression of CSCs to promote their self-renewal, invasion, and metastasis. Also, Cripto-1 expression is described to be associated with NANOG or OCT4 and *vice versa*. Cripto-1 can be considered a functional CSCs marker. Recent evidence also supports the role of epigenetics in the maintenance of CSCs. Histone modification patterns, particularly histone acetylation conducted *via* histone acetyltransferases and histone deacetylases (HDACs), perform necessary transcriptional regulation (Dong, 2016). In this regard, aberrantly expressed class III histone deacetylases (HDACs), also known as sirtuins (SIRTs), are also described to possess a significant role in cancer stem-ness, especially SIRT1 as the best-described SIRT (O’Callaghan and Vassilopoulos, 2017). In addition, SIRT3 and SIRT6 are also crucial for the self-renewal and tumour-initiation functions of CSCs (Sharma et al., 2022). In addition, the expression of stem cell-associated histone modifier genes, such as *EZH2* contributes to self-renewal and BCSCs expansion (Jia et al., 2016). Other markers of breast cancer invasiveness and metastatic potential related to cancer stem-ness include chemokine receptor type 4 (CXCR4), which is widely expressed in various cancer types and is closely associated with migration and metastasis in breast cancer and mucin 1 (MUC1) that acts *via* binding to EGFR and β-catenin. Increased MUC1 also correlates with the invasiveness and metastatic potential of breast cancer. Moreover, epithelial cell adhesion molecule (EpCAM) is another marker of CSCs, which also acts on the proliferation of cancer cells (Wang et al., 2018).

In conclusion, BCSCs are responsible for cancer recurrence and treatment failure. Therefore, CSCs targeting and their elimination can be considered the most crucial approach for effective cancer management (Mauro-Lizcano et al., 2022).

## 3 Current research on flavonoids targeting CSCs with the potential to combat resistance

Flavonoids are polyphenols with a basic skeleton (C6-C3-C6) with two phenolic (A and B) rings and a heterocyclic pyran (C) ring (Panche et al., 2016; Liskova et al., 2020c; Koklesova et al., 2021; Mazurakova et al., 2022b; Shen et al., 2022). Based on the chemical structure, oxidation level, and substitution pattern of the C ring, flavonoids are divided into several sub-classes, including flavonols, flavones, isoflavones, anthocyanidins, flavanones, and flavan-3-ols (Liskova et al., 2021). Chalcones also belong to the flavonoid family; however, the chalcone skeleton is characterized as the initial intermediate in the biosynthesis of all flavonoids (Ninomiya et al., 2013) and is defined as the flavonoid precursor (Gao et al., 2020; Ouyang et al., 2021). Chalcones are chemically characterized by the presence of two aromatic rings and the absence of C ring and are, therefore, known as open-chain flavonoids (Ninomiya et al., 2013; Panche et al., 2016). Chalcones and flavanones, dihydrochalcones, and aurones (the isomeric of flavones) represent minor flavonoids (Ninomiya et al., 2013). Our review provides a detailed discussion on the evidence of the effects of flavonoids against BCSCs with the potential to combat the resistance of cancer cells to standard therapeutic strategies due to the close association between cancer stem-ness and drug resistance. Table 1 provides a detailed overview of flavonoids discussed in this review with their sub-classification within the class of flavonoids, chemical formula, and examples of primary natural sources.

**TABLE 1:** Classification, chemical formula, and natural sources of selected flavonoids.

Flavonoid	Chemical formula	Classification	Examples of natural sources	References
Quercetin	3,3′,4′,5,7-pentahydroxyflavone	Flavonol	Berries, grapes, citruses, and black tea	Kim and Park (2018), Salehi et al. (2020)
Kaempferol	3,5,7-trihydroxy-2-(4-hydroxyphenyl)-4H-chromen-4-one	Flavonol	Grapes, tea, broccoli, and ginkgo biloba leaves	Calderón-Montaño et al. (2011), Kim and Park (2020)
Genistein	4′,5,7-trihydroxyisoflavone	Isoflavone	Soy, broccoli, cauliflower, sunflower, barley meal	Tuli et al. (2019), Sharifi-Rad et al. (2021)
Naringenin	4′,5,7-trihydroxy flavanone	Flavanone	Citrus fruits, bergamot, tomatoes	Salehi et al. (2019)
Apigenin	4′,5,7-trihydroxyflavone	Flavone	Predominantly found as glycosylated in parsley, celery, citruses, and herbs	Salehi et al. (2020)
Baicalein	5,6,7-trihydroxy-2-phenyl-4H-1-benzopyran-4-one	Flavone	*Scutellaria baicalensis* Georgi	Bie et al. (2017), Liskova et al. (2020c)
Chrysin	5,7-dihydroxy-2-phenyl-4H-chromen-4-one	Flavone	Honey, propolis, and passion fruit	Naz et al. (2019)
Luteolin	3,4,5,7-tetrahydroxyflavone	Flavone	Celery, carrots, or parsley	Imran et al. (2019)
EGCG	Epigallocatechin-3-gallate	Flavanol	Green tea, black tea	Nagle et al. (2006), Eng et al. (2018)
Ampelopsin	(2R,3R)-3,5,7-trihydroxy-2-(3,4,5-trihydroxyphenyl)-2,3-dihydrochromen-4-one	Flavanol	Medicinal plants of *Ampelopsis* species or *Hovenia dulcis* (Japanese raisin tree)	Zhou et al. (2014), Han et al. (2021), Li and Xiao (2021), Truong et al. (2021)
Xanthohumol	3′-[3, 3-dimethyl allyl]-2′,4′,4-trihydroxy-6′-methoxychalcone	Chalcone	Hops (*Humulus lupulus* L.)	Harish et al. (2021)
Cardamonin	2′,4′-dihydroxy-6′-methoxychalcone	Chalcone	Petals, fruit, leaves, bark, and roots of plants, frequently of the family Zingiberaceae	Voon et al. (2017), Nawaz et al. (2020)

### 3.1 Flavonoids targeting BCSCs

The effects of flavonoids on BCSCs are evaluated mainly in experimental, especially *in vitro* research. Several cell lines represent a suitable breast cancer cell model source to study BCSCs. For example, MDA-MB-231 is a TNBC cell line characterized by mesenchymal phenotype, with more than 90% of the total population expressing CD44^+^/CD24^low/−^ (Hero et al., 2019). MDA-MB-468 and MDA-MB-436 represents other TNBC cell lines frequently used to study stem-cell characteristics (Li et al., 2018aLi et al., 2018a). In addition, MCF-7 is a luminal subtype breast cancer cell line (Ambrose et al., 2022) also frequently utilized to study stem-ness properties of breast cancer.

The search on most recent evaluations of the anticancer effects of flavonoids provide significant evidence on the efficacy of quercetin, kaempferol, apigenin, genistein, and naringenin (Fan et al., 2013; Li et al., 2018a; Wang et al., 2018; Hermawan et al., 2021; Sharma et al., 2022) [as the most widely naturally distributed and studied flavonoids (Calderón-Montaño et al., 2011; Kim and Park, 2020; Salehi et al., 2020)] to target BCSCs, which are closely associated with breast cancer aggressiveness, recurrence, and therapy resistance (Wang et al., 2018; Tsai et al., 2021). Quercetin is present mainly as glycoside rather than aglycone. Importantly, quercetin shows higher bioavailability than other phytochemicals (Kim and Park, 2018; Salehi et al., 2020). Quercetin inhibited proliferation, clonal expansion, mammosphere formation, and migration of MDA-MB-231 of CD44^+^/CD24^−^ phenotype (BCSCs populations). Mechanistically, quercetin downregulated ALDH1, EpCAM, CXCR4, and MUC1, which are all reported as potential targets for BCSCs treatment (Wang et al., 2018) (Salehi et al., 2020).

Both kaempferol and apigenin show significant anti-stemness capacity as demonstrated *via* suppressed colony-forming potential and mammosphere formation in MDA-MB-468 cells *in vitro*. Moreover, mechanisms beyond anti-CSCs effects of kaempferol and apigenin are associated with targeting SIRT3 and SIRT6 (as demonstrated *via* network pharmacology), while molecular docking analysis supported the capacity of these two flavonoids as potential anti-SIRT agents to target CSCs (Sharma et al., 2022).

Moreover, the capacity of apigenin to suppress stem cell properties was demonstrated *via* decreased CD44^+^/CD24^−^ subpopulation and mammospheres thus indicating reduced self-renewal capacity of MDA-MB-231 and MDA-MB-436 cells *in vitro* and suppressed tumour-initiating properties *in vivo*. Apigenin dose dependently inhibited YAP/TAZ and thus reversed malignant phenotype and disrupted YAP/TAZ-TEADs interactions, which was previously demonstrated to be indispensable for TAZ in maintaining stem cell traits in breast cancer cells. The authors concluded that apigenin showed potent anticancer efficacy at least partially *via* YAP/TAZ inhibition and decreased expression of target genes *CTGF* and *CYR61* (Li et al., 2018a).

Moreover, significant action against BCSCs is attributed to genistein, a major soy isoflavone and naringenin, a precursor of genistein. Specifically, genistein suppressed BCSC *in vitro* demonstrated *via* reduced number and size of mammospheres and CD44^+^CD24^–^ population in genistein-treated MCF-7 cells. Moreover, genistein significantly reduced tumour weight and BCSC demonstrated *via* ALDH when compared with control group in a xenograft model of MCF-7 cells in nude mice. Above all, the authors concluded the potent anti-BCSCs capacity of genistein to be mediated *via* downregulation of Hedgehog-Gli1 signalling pathway (Fan et al., 2013). In addition to Hedgehog, aberrant signalling pathways closely related to cancer stem cells include also Notch and Wnt/β-catenin (Liskova et al., 2019).

Furthermore, Hermawan et al. (2021) recently evaluated the potential therapeutic target of naringenin in BCSCs inhibition using bioinformatics study and 3D tumour-sphere *in vitro* modelling in breast cancer mammospheres. Among other results, naringenin inhibited mammospheres and colony formation, migration, and EMT in mammospheres. Naringenin showed the potential to inhibit BCSCs *via* the ability to upregulate P53 and ERα mRNA (that were found to be downregulated in mammospheres).

### 3.2 Flavonoids targeting BCSCs: Evidenced effects on resistance

The development of resistance to standard therapeutic strategies represents a severe obstacle of breast cancer management (Mazurakova et al., 2022a). For example, the enrichment of BCSCs following chemotherapy prevents effective management of breast cancer patients. Therefore, the eradication of BCSCs by the combination of standard cytotoxic drugs and substances specifically targeting CSCs is necessary for successful treatment (Jia et al., 2016). Flavonoids show a potent capacity to target BCSCs and therapeutic resistance, as evidenced predominantly *in vitro* and *in vivo* models of breast cancer.

#### 3.2.1 Flavonols—Kaempferol and quercetin

Kaempferol alone or combined with a P-gp inhibitor verapamil (at a specific concentration to induce synergism) impeded pH-dependent tumour-sphere formation *in vitro*/*ex vivo*. Besides, tumour acidosis promotes CSCs phenotype and the development of therapeutic resistance. Moreover, kaempferol alone or with verapamil repressed the expression of CD44, OCT4, NANOG, and MDR1, disrupting the physical association of CD44, NANOG, and MDR1. Moreover, the dosage of this experimental candidate drug system revealed no genotoxic effects on normal breast tissues (Nandi et al., 2022a).

Similarly, kaempferol downregulated CD44, ALDH1, NANOG, MDR1, Ki67, BCL2, p53 and upregulated caspase-3 *ex vivo*. The authors describe the evidence on the association between p53 inactivation and the support of stem-ness and MDR1 upregulation together with the expression of other genes, thus ensuring chemo-tolerance of cancer patients *via* NF-κB mediated and p53 related pathways. This observation was also proved by the results on analysed *ex vivo* grown breast tumours *via* a high degree of co-expression of p53 and CD44, Ki67, ALDH1, NANOG, MDR1, BCL2, and NF-κB, thus supporting the potential of kaempferol to target specific oncogenic markers in a biomarker-driven therapeutic strategy (Nandi et al., 2022b).

Another study evaluated the impact of quercetin on MDR in breast cancer *in vitro*. The initial observations described overexpressed YB-1, P-gp and CD44^+^/CD24^−/low^ phenotype (BCSCs) in doxorubicin-resistant MCF-7 (MCF-7/dox) cells. As stated before, YB-1 translocation significantly contributes to P-gp overexpression and CD44^+^/CD24^−/low^ phenotype. Eventually, quercetin combined with docetaxel also promoted the apoptosis of MCF-7 and MCF-7/dox cells. Moreover, combined treatment of quercetin and chemotherapeutics (docetaxel, paclitaxel, and vincristine) suppressed YB-1 nuclear translocation resulting in P-gp inhibition and eliminated CD44^+^/CD24^−^ cells (thus eliminating BCSCs) in both analysed cell lines. Above all, quercetin promoted the anticancer effectivity of analysed chemotherapeutics, inhibited chemotherapeutics-induced YB-1 nuclear translocation, and promoted intracellular accumulation of docetaxel in MCF-7/dox (Li et al., 2018b).

#### 3.2.2 Flavones—Baicalein, chrysin, and luteolin

Baicalein showed efficacy as a sensitizer to overcome therapy resistance in TNBC cells *in vitro*. Koh et al. (2019) established treatment resistant TNBC cell model *via* irradiation of parental MDA-MB-231 cells (MDA-MB-231/IR); eventually, established chemo- and radio-resistant cells showed promoted invasion, migration, CSCs characteristics as well as enriched NF-κB pathway, TNF pathway, and Toll-like receptor (TLR) pathway. Moreover, transcriptome analysis revealed differential expression of several genes involved in radio- and chemo-resistance, including *IFIT2*. However, baicalein suppressed stem cell-like properties in chemo- and radio-resistant MDA-MB-231/IR cells and reversed *IFIT2* expression.

Several anticancer therapies target the receptor tyrosine kinases (TRKs), which contribute to tumorigenesis through downstream signalling. Epidermal growth factor receptor (EGFR) is a tyrosine kinase that is overexpressed in numerous tumours and is involved in cancer cell proliferation. However, despite reduced side effects when compared with chemotherapy, EGFR inhibitors show low clinical activity. Thus, there is a need for the identification of more effective substances to target EGFR (Manupati et al., 2017). As demonstrated by Manupati et al. (2017), TNBC cancer cells (specifically MDA-MB-231) are characterized by higher EGFR expression when compared with a luminal cell line (MCF-7 cells). The authors also observed higher EGFR expression in BCSCs than in breast cancer cells. CHM-09, synthetized analogue of chrysin (isolated from *Oroxylum indicum* bark) with improved binding affinity to EGFR, effectively suppressed EGF-induced migration, mammosphere formation, and tri-lineage differentiation of CD24^−^/CD44^+^ breast CSCs, accompanied a reversal of EMT or the induction of MET in breast CSCs by the attenuation of EGFR signalling. The authors concluded increased responsiveness to chemotherapeutics when simultaneously treated with doxorubicin and EGFR inhibitors. Thus, EGFR represents a target of anticancer strategies due to its high expression in BCSCs (Manupati et al., 2017).

In addition, luteolin at various concentrations inhibited stemness-related proteins, ALDH1 activity, sphere formation as well as Nrf2, SIRT3, Cripto-1, and HO-1 in MDA-MB-231 cells and promoted their chemosensitivity to taxol. In conclusion, luteolin diminished the hallmarks of BC stem-ness by Nrf2-mediated pathway (Tsai et al., 2021).

#### 3.2.3 Flavanols—EGCG and ampelopsin

Recent research supports the role of lipid metabolism in BCSCs promotion and chemo-resistance. An enzyme fatty acid synthase (FASN) is involved in *de novo* synthesis of the most abundant fatty acid palmitate. Indeed, several cancer types overexpress FASN, while the blockage of FASN resulted in the inhibition of cancer progression, overcoming resistance, and promoting the effectiveness of chemotherapy *in vitro* and *in vivo*. The association between FASN and drug resistance is based on the new phospholipid synthesis for membrane renovation and plasticity (Giró-Perafita et al., 2019). FASN has been shown to be overexpressed in most tumour tissues of a cohort of 100 TNBC patients (Giró-Perafita et al., 2017; Giró-Perafita et al., 2019). The flavanol epigallocatechin-3-gallate (EGCG) is the main catechin of green tea (Nagle et al., 2006). Giró-Perafita et al. (2019) studied the effects of EGCG and its synthetic derivatives on the FASN inhibition in BCSC-enriched populations resistant to doxorubicin (MDA-MB-231DXR) and paclitaxel (MDA-MB-231PTR). Eventually, the mammosphere-forming capacity of tested TNBC cells *in vitro* was significantly inhibited after the treatment with G28, the derivative of EGCG.

Recently, Truong et al. (2021) evaluated the effects of ampelopsin [also known as dihydromyricetin (Weng et al., 2017)] in TNBC cell line MDA-MB-231/IR, characterized by better stem-ness features, chemo-resistance, and radio-resistance when compared with parental MDA-MB-231 cells. Specifically, ampelopsin at different concentrations inhibited stem-ness features mammosphere formation, CD44^+^/CD24^−/low^ and ALDH^+^ populations, invasion, migration, and EMT markers in resistant breast MDA-MB-231/IR cells. Moreover, ampelopsin inhibited oxidative phosphorylation in the same cell line. Indeed, CSCs show flexibility in switching between glycolysis and oxidative phosphorylation; however, recent evidence describes enhanced oxidative phosphorylation to maintain the stemness of CSCs and resistance to DNA damage. Also, the authors concluded that ampelopsin prevented TNF-α/NF-κB signalling in breast CSCs (Truong et al., 2021).

#### 3.2.4 Chalcones—Xanthohumol and cardamonin

Similarly, a prenylated chalcone xanthohumol enhanced the sensitivity of doxorubicin-resistant MCF-7/ADR cells to docetaxel demonstrated *via* significantly reduced IC_50_ value when compared with docetaxel alone. Also, when compared with the effects on parental cells sensitive to docetaxel, the authors concluded that the synergistic effects of xanthohumol and docetaxel were not as apparent as in docetaxel-resistant cells. In addition, xanthohumol at different concentrations reduced SP cell fractions, alleviated clonogenicity, suppressed mammosphere formation and migration in a concentration-dependent manner, reduced stem cell markers, including CD44^+^/CD24^−^ populations, ABCG2 and Notch1 in doxorubicin-resistant MCF-7/ADR cells; indeed, these effects were accompanied by inhibited viability, induced apoptosis, and cell cycle arrest (Liu et al., 2016).

Cardamonin, a cardamom-derived chalcone reduced colony-forming capacity, CD44^high^/CD24^−/low^ subpopulations, and expression of CSC-associated genes, and stem cell-associated modifier genes in chemotherapy-enriched breast cancer stem-like SUM-190 cells. These results highlight the potential of cardamonin to target BCSCs after chemotherapy completion; moreover, cardamonin seems to act on diminishing CSC properties rather than reducing CSC populations, thus promoting the conversion of BCSCs into non-CSCs. In addition, the combinatory treatment with chemotherapy and cardamonin mitigated CSCs phenotype. Cardamonin also abrogated inflammatory cytokines closely related to CSCs development and diminished the activation of NF- κB, IκBα, and Stat3 *in vitro*. Moreover, cardamonin combined with doxorubicin enhanced the efficiency of chemotherapy *in vivo via* suppressing tumour growth and reducing CSCs, eliminated doxorubicin-enriched CSCs, and diminished doxorubicin-upregulated stemness genes (*ALDH1*, *SOX2*, *OCT4*) (Jia et al., 2016).

Table 2 provides a detailed overview of the above-discussed flavonoids that exert either the potential to combat BCSCs or their capability to target BCSCs with evidenced effects on therapeutic resistance as the crucial obstacle in the effective management of breast cancer.

**TABLE 2:** Flavonoids targeting BCSCs to combat therapeutic resistance.

Flavonoid	Analysed concentration/dosage (duration)	Study details	Results	Effect	References
*Flavonoids targeting BCSCs*					
Quercetin	12.5, 25, 50, 100, and 200 μM (12—96 h)	MDA-MB-231 cells with CD44^+^/CD24^−^ phenotype	↓ proliferation, clonal expansion, mammosphere formation, migration	Suppressed stem-cell properties	Wang et al. (2018)
			↓ ALDH1, CXCR4, MUC1, EpCAM		
Kaempferol and apigenin	Kaempferol—12.5 and 25 μg/mL; Apigenin—6 and 12 μg/mL	MDA-MB-468	↓ colony-forming potential	Suppressed stemness	Sharma et al. (2022)
			↓ mammosphere formation		
		Network pharmacology, molecular docking, molecular dynamics analysis	↓ SIRT3 and SIRT6	Anti-SIRT effects targeted against CSCs	
Apigenin	5—64 μM (24—72 h)	MDA-MB-231 and MDA-MB-436 *in vitro*	↓ CD44^+^/CD24^−^ subpopulation and mammospheres (*in vitro*)	Suppressed stem cell properties	Li et al. (2018a)
			↓ YAP/TAZ (→ reversed malignant phenotype)		
			Disrupted YAP/TAZ-TEAD interaction		
	20 μM (48 h)	Apigenin-treated MDA-MB-231 cells injected into female BALB/c mice	↓ tumour-initiating properties (*in vivo*)		
Genistein	5—30 μM	MCF-7	↓ mammosphere formation	Suppressed stem cell properties	Fan et al. (2013)
			↓ CD44^+^/CD24^−^		
			↓ Hedgehog-Gli1		
	Intraperitoneal injection with control or 20 and 50 mg/kg genistein respectively (daily for 2 weeks)	MCF-7 cells injected into mouse mammary fat pad (female nude mice)	↓ tumour weight		
			↓ ALDH		
			↓ Hedgehog-Gli1		
Naringenin	3D tumour-sphere *in vitro* modelling in breast cancer mammospheres (100 μM)	MCF-7 cells cultured to generate mammospheres and bioinformatics	↓ mammospheres; colony formation, migration, and EMT in mammospheres	Suppressed stem cell properties	Hermawan et al. (2021)
			↑ P53 and ERα mRNA		
*Flavonoids targeting BCSCs—evidenced effects on resistance*					
Kaempferol alone or in combination with verapamil	Kaempferol—224.51 μM; Verapamil—5 μM; Combined kaempferol and verapamil—104.8 μM and 5 μM	Stem cell-enriched MDA-MB-231 cell line (*in vitro*)	↓ pH-dependent tumour-sphere formation	Suppression of CD44-NANOG-MDR1 associated chemo-resistance	Nandi et al. (2022a)
			↓ CD44, OCT4, NANOG, MDR1		
		Primary tumours isolation and stem cell-enriched ex vivo culture (primary tumour samples from patients with neoadjuvant chemotherapy)	↓ physical attenuation of CD44 with NANOG and MDR1		
			↓ CSCs markers (CD44, OCT4, NANOG, MDR1)		
Kaempferol	224.51 μM (48 h)	*Ex vivo*-grown breast tumours derived from post-neoadjuvant chemotherapy patients	↓ CD44, ALDH1, NANOG, MDR1, Ki67, BCL2, p53	Downregulation of p53-induced stemness	Nandi et al. (2022b)
			↑ Caspase-3		
			High degree of co-expression of p53 and CD44, Ki67, ALDH1, NANOG, MDR1, BCL2, and NF-κB		
Quercetin or quercetin combined with chemotherapeutics	0.7 μM (24 h)	MCF-7/dox cells (*in vitro*)	↑ anticancer effects of docetaxel, paclitaxel, and vincristine	MDR reverse	Li et al. (2018b)
			↓ YB-1 nuclear translocation, P-gp and CD44^+^/CD24^−^ (BCSC), (quercetin acting synergistically with docetaxel, paclitaxel, vincristine)		
Baicalein	10—80 μM (6—24 h)	MDA-MB-231/IR	↓ mammospheres, side population	Suppressed stem cell-like properties in chemo- and radio-resistant TNBC	Koh et al. (2019)
			↓ ABCG2, OCT3/4, and CD44^high^CD24^low^ population		
			Reversed IFIT2		
CHM-09, synthesized analogue of chrysin	0.1—10 μM (12—24 h)	MDA-MB-231 cells *in vitro*	↓ phosphorylation of EGFR and downstream signalling Akt, ERK, STAT3	Increased responsiveness to chemotherapy when combined with EGFR inhibitors	Manupati et al. (2017)
			↓ mammosphere formation and migration		
			Loss of mesenchymal phenotype (↓ vimentin, fibronectin)		
			Gain of epithelial phenotype (↑ E-cadherin)		
Luteolin	0.5—2 μM (48 h)	MDA-MB-231	↓ ABCG2, Nanog, Oct4, CD44	Suppressing stem-cell properties; Increasing chemosensitivity to taxol	Tsai et al. (2021)
			↓ antioxidant proteins (HO-1, Nrf2, Cripto-1, SIRT3)		
			Regulation of BC stemness *via* Nrf2		
EGCG derivative - G28	50 μM	MDA-MB-231 resistant to doxorubicin and paclitaxel	↓ mammosphere formation	Rational for FASN suppression as a strategy to target TNBC	Giró-Perafita et al. (2019)
Ampelopsin	3.125—200 μM (24 h)	MDA-MB-231/IR	↓ CSCs features (↓ mammosphere formation, ↓ CD44^+^/CD24^−^/low and ALDH-positive populations, ↓ CD44, β-catenin, KLF4)	Suppressing stem-cell properties	Truong et al. (2021)
			↓ migration, invasion		
			↓ EMT markers: Snail, Slug, MMP2, ↑ E-cadherin		
			↓ OXPHOS		
			↓ TNF-α/NF-κB signalling		
			↑ cytotoxicity		
Xanthohumol	2.5—40 μM (24—72 h)	MCF-7/ADR	↑ sensitivity to docetaxel (synergism of docetaxel and xanthohumol)	Suppression of stemness and increase in sensitivity to docetaxel	Liu et al. (2016)
			↓ SP		
			↓ mammosphere formation		
			↓ clonogenicity		
			↓ CSC markers (CD44^+^/CD24^−^ populations; ↓ Notch1, ABCG2)		
			↓ viability; ↑ apoptosis; ↑ cell cycle arrest		
Cardamonin	7.5 μM	Chemotherapy-enriched breast cancer stem-like SUM-190, MCF-7, Cama-1 cells (*in vitro*)	↓ colony-formation	Targeting BCSCs after chemotherapy; suppressing CSCs	Jia et al. (2016)
			↓ CD44^high^/CD24^−/low^		
			↓ *ALDH1*, *c-MYC*, *SOX2*, *OCT4*		
			↓ *SMYD3*, *SETDB1*, *EZH2*		
			↓ NF- κB, IκBα, and Stat3		
			↓ upregulation of ALDH1, c-MYC, and OCT4 proteins, CD44^high^/CD24^−/low^ subpopulations, *ALDH1*, *SOX2*, *c-MYC*, *OCT4*, *NANOG*, and *EZH2*, *SETDB1*, *SMYD3*	Mitigated CSC phenotype and enhanced the efficiency of chemotherapy	
			↓ IL-6, IL-8, MCP-1	Mitigated inflammatory cytokines associated with CSCs development	
Cardamonin combined with doxorubicin	Injected intraperitoneally with vehicle, doxorubicin (6 mg/kg on days 13, 18, and 23), cardamonin (25 mg/kg once every other day from days 13) or a combination	Athymic nude mice transplanted with SUM-190 cells (*in vivo*)	↓ tumour growth	Enhanced efficiency of chemotherapy	
			↓ doxorubicin-enriched CSCs		
			↓ doxorubicin-upregulated stemness genes (*ALDH1*, *SOX2*, *OCT4*)		

Abbreviations: ABCG2, ATP-binding cassette transporter G2; ALDH1, aldehyde dehydrogenase 1; BC, breast cancer; BCSCs, breast cancer stem cells; CD, cluster of differentiation; CSCs, cancer stem cells; CXCR4, chemokine receptor type 4; EMT, epithelial to mesenchymal transition; EpCAM, epithelial cell adhesion molecule; HO-1, heme oxygenase 1; IL, interleukin; MDR, multidrug resistance; MMP, matrix metalloproteinase; MUC1, mucin 1; Nrf2, nuclear factor erythroid 2-related factor 2; OXPHOS, oxidative phosphorylation; RNA, ribonucleic acid; SIRT, sirtuins; TNBC, triple-negative breast cancer.

### 3.3 Plant extracts rich in flavonoids

In addition to isolated flavonoids, a mixture of numerous phytochemicals within the whole plant also exert potent anticancer effects *via* their synergistic or additive action (Kubatka et al., 2015; Kapinova et al., 2017; Kubatka et al., 2017; Kubatka et al., 2020a; Kubatka et al., 2020b; Liskova et al., 2020c). For example, a recent study demonstrated the potent anticancer effectivity of polyphenols isolated from *Artemisia annua* L. to inhibit CSC markers, β-catenin and matrix metalloproteinase 9 (MMP-9), all of which were overexpressed in radio-resistant MDA-MB-231 cells *in vitro* (Ko et al., 2020). Moreover, extract of *Viola odorata* characterized by numerous phytochemicals, including flavonoids luteolin, quercetin, apigenin, or kaempferol, showed potent anticancer effects on breast cancer cells (MCF-7, SKBR3) and derived BCSCs. Specifically, the extract of *Viola odorata* suppressed colony formation, migration, and invasion of derived mammospheres more than in corresponding breast cancer cells. Moreover, the extract inhibited tumorigenicity in evaluated breast cancer cells and derived mammospheres in chicken embryos *in vivo* (Yousefnia et al., 2020). Woo et al. (2017) observed that CSCs derived from breast cancer cells MCF-7 with CD44^high^/CD24^low^ phenotype form mammospheres and overexpress Nrf2 when compared with parental MCF-7 cells. Chestnut is rich in numerous polyphenols, including flavonoids (Tuyen et al., 2017). Chestnut (*Castanea crenata*) leaf extract suppressed nuclear translocation of Nrf2, suppressed ARE-luciferase activity, and reduced protein expression of Nrf2-downstream gene HO-1 in MCF-7 derived CSCs. Chestnut leaf extract also suppressed colony formation of CSCs, while more effective inhibition of clonogenicity was observed after the combinatory treatment with paclitaxel. Therefore, CSCs treatment with chestnut leaf extract combined with paclitaxel showed more efficacy in reducing the viability of CSC when compared with paclitaxel alone; however, such combinatory treatment showed no significant effects on MCF-7 cells (Woo et al., 2017). Also, solvent fractions of selected medicinal plants (*Vernonia leopoldi*, *Clematis simensis*) (Tuasha et al., 2020), characterized by the presence of numerous phytochemicals, including flavonoids (Teshome et al., 2022; Tuasha et al., 2022), showed a potent capacity to reduce ALDH^+^ subpopulations of JIMT-1 cells, and/or reduce colony formation, and/or reduced migration, and/or inhibit TNF-α-induced NF-κB nuclear translocation (Tuasha et al., 2020). Many other plant extracts show significant capacity to suppress CSCs in breast cancer models, for example, pomegranate extract targeting BCSCs and EMT (Nallanthighal et al., 2017) or hexane extract of *Garcinia quaesita* fruits inducing apoptosis of BCSCs isolated from MDA-MB-231 cells (Colamba Pathiranage et al., 2021) with the potential to increase the effectiveness of therapeutics.

**FIGURE 2:**
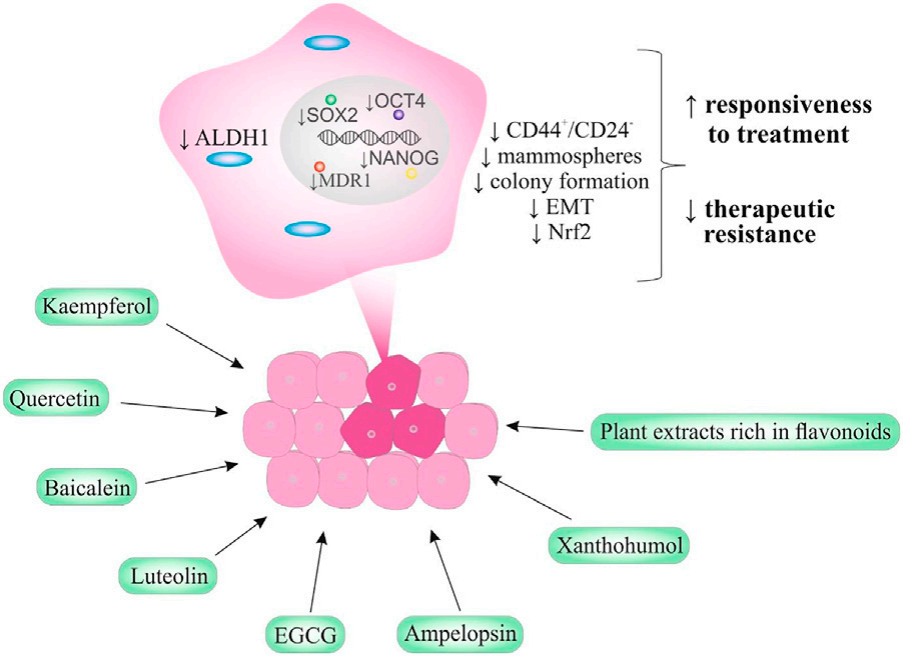
Flavonoids targeting BCSCs (dark pink)—Potential to combat therapeutic resistance.

In conclusion, above discussed preclinical evidence on the effects of flavonoids against BCSCs can represent an essential cornerstone for further clinical evaluations. Figure 2 provides an illustrated overview of BCSCs markers/characteristics that are associated with the resistance of breast cancer to therapeutics and are most frequently demonstrated to be modulated by flavonoids in most current *in vitro* and *in vivo* studies.

## 4 Flavonoids: Limitations and potential in clinical evaluations

Here we provided a detailed overview of current evidence on the effects of flavonoids to target BCSCs to overcome breast cancer resistance to standard therapeutics. Above discussed study results focused primarily on *in vitro* analyses of the anticancer efficacy of flavonoids (Jia et al., 2016; Woo et al., 2017; Truong et al., 2021; Nandi et al., 2022b; Sharma et al., 2022). However, the discussion on the advantages and limitations of flavonoids as well as the currents state of their evaluation in clinical studies is essential to provide a comprehensive review anti-cancer effects of flavonoids targeted against BCSCs.

Naturally occurring phytochemicals are advantageous due to their pluripotency, minimal or no side effects, and cost-effectiveness (Liskova et al., 2020c; Koklesova et al., 2021; Liskova et al., 2021; Samec et al., 2021). However, the anticancer capability of *in vivo* or in a clinical setting is associated with numerous complications. Firstly, flavonoids are characterized by low bioavailability due to their characteristics and extensive metabolism (Liskova et al., 2020c). For example, dihydromyricetin is generally associated with poor bioavailability due to the hydrophilic character (Li et al., 2017). Flavonoid metabolites are generally associated with reduced bioactivity when compared with parental compounds (Thilakarathna and Rupasinghe, 2013). Flavonoids present most abundantly in our diet may not reach target organs. On the contrary, several flavonoid metabolites are associated with more potent bioactivity than their precursors (Murota et al., 2018; Liskova et al., 2020c). The utilization of flavonoids as health-promoting agents also highly depends on individual characteristics such as age, sex, habitual diet, genotype, and gut microbiome, which plays an essential role in the absorption, distribution, tissue exposure, and elimination of phytochemicals (Liskova et al., 2020c). Still, the current scientific community also analyses the approaches to improve the bioavailability and utilization of flavonoids against cancer, for example, *via* absorption enhancers, structural transformation, carrier complexes, or nanotechnology, among others (Thilakarathna and Rupasinghe, 2013; Zhao et al., 2019).

Despite the peculiarities associated with limited bioavailability of phytochemicals, current evidence on the evaluation of the effects of phytochemicals in clinical settings still provide at least promising results (Mazurakova et al., 2022a; Mazurakova et al., 2022c). Lazzeroni et al. (2017) demonstrated that oral administration of a lecithin formulation of a caffeine-free green tea catechin extract (Greenselect Phytosome) increases EGCG bioavailability, while EGCG can be detectable in breast tumour tissues of early breast cancer patients. Also, free EGCG plasma levels significantly correlated with Ki67 decrease in tumour tissues (Lazzeroni et al., 2017). Above all, as reviewed previously, phytochemicals showed potential in the prevention of breast cancer in terms of dietary patterns and breast cancer risk or in the mitigation of side effects of cancer therapies (for example, silymarin reducing radiodermatitis or EGCG reducing acute skin damage induced by radiation) (Zhu et al., 2016; Karbasforooshan et al., 2019; Mazurakova et al., 2022a). Moreover, flavonoids showed efficacy in enhancing the effectiveness of conventional therapeutic strategies demonstrated in clinical studies, for example, EGCG enhanced radiotherapy in breast cancer patients and affected cell proliferation, invasion, and cell cycle among others (Zhang et al., 2012). In addition, green tea extract, Polyphenon E resulted in non-significant decrease of VEGF by 11.5% at 2 months (*p* = 0.02) and 13.9% at 4 months (*p* = 0.05) in hormone-receptor negative breast cancer patients (Crew et al., 2015). In addition, the overweight after breast cancer treatment can increase the risk of recurrent disease; however, decaffeinated green tea taken for 6 months was associated with slightly reduced body weight and improved HDL and glucose homeostasis in overweight breast cancer survivors (Stendell-Hollis et al., 2010). On the contrary, Wang et al. (2009) demonstrated no association of dietary intake of flavonols (quercetin, kaempferol, myricetin), flavones (apigenin and luteolin), or flavonoid-rich food and incidence of cancer in middle-aged and older women. Therefore, despite above-discussed evidence, the potential of flavonoids in clinical research cannot be definitely confirmed. As an example, despite that epidemiological studies predominantly highlight the potential of soy (that contains isoflavones genistein and daidzein) in breast cancer prevention, the effects of soy on breast cancer is not clearly understood yet (Shike et al., 2014). A six-months intervention of mixed soy isoflavones in healthy, high-risk adult Western women did not result in reduced epithelial proliferation thus suggesting inefficacy in breast cancer prevention (Khan et al., 2012). On the contrary, the consumption of soy products is associated with lower risk of breast cancer in BRCA mutations carriers (Ko et al., 2013) or with reduced fibroglandular breast tissue, a breast cancer risk marker (Lu et al., 2022). Moreover, as demonstrated in a population-based study on TNBC cohort, long-term pre-diagnosis consumption of soy, a rich source of isoflavones, may lead in increased expression of tumour suppressors and decreased expression of oncogenes in breast tumour tissue (Guo et al., 2016).

Above all, here we provided an overview of the available results of the utilization of flavonoids in clinical settings, especially in enhancing the effectiveness or mitigating the side effects of conventional therapy (Mazurakova et al., 2022a). Still, despite proved anti-cancer potential of flavonoids (or phytochemicals in general) in affecting each of the multistep process of carcinogenesis in experimental *in vitro* and *in vivo* settings (Kapinova et al., 2017; Jasek et al., 2019; Liskova et al., 2020a; Liskova et al., 2020c; Koklesova et al., 2021; Koklesova et al., 2021; Liskova et al., 2021; Samec et al., 2021), the research evidence on the potential of flavonoids in clinical studies is generally limited, while the clinical evaluation of potent effectiveness of flavonoids in targeting BCSCs is not available. Nevertheless, the precise clinical evaluation of the anti-cancer effectiveness of phytochemicals is highly required for improvement of cancer management and a shift from reactive to 3PM medicine. BCSCs represents a crucial target in cancer management due to their role in cancer initiation, progression, invasiveness, metastasis, and therapeutic resistance. The potential utilization of flavonoids targeting BCSCs requires precise evaluation of multiple approaches, including the precise mechanisms of action of flavonoids in specific conditions as well as the characteristics of the individual patient.

## 5 Conclusion and future outlooks

The appropriate management of breast cancer requires strategies to be beneficial for the patient and society as a whole. Primary care represents the protection of individuals against cancer development. Secondary care is associated with protection against the development of metastatic disease and tertiary care is targeted against cascading complications related to palliative care and chronic disease management (Liskova et al., 2020c). The evidence in detail discussed in this review supports flavonoids’ great potential, especially in secondary care of breast cancer patients, due to the capacity of flavonoids to combat resistance and thus could potentially provide more beneficial therapeutic outcomes for breast cancer patients. The non-responsiveness of cancer patients to treatment strategies or relapse of the disease after initial responses or after years of remission urgently highlight the need of novel strategies to be identified. The incidence of breast cancer is alarming, and the decreasing age at the diagnosis of breast cancer further support the need to improve cancer management in the framework of 3PM.

Above presented research data are considered highly beneficial for innovative strategies in the framework of 3P medicine promoting predictive approach, targeted prevention, and personalisation of medical services (Mazurakova et al., 2022a). Relevant clinical applications can be exemplified by

- Hypoxia-induced breast cancer stem cells (Zhang et al., 2016). 3PM approach may consider relevant phenotyping such as patients stratified by Flammer syndrome demonstrating evident microcirculation deficits associated with systemic ischemia-reperfusion events and particularly aggressive breast cancer with poor outcomes (Bubnov et al., 2017; Golubnitschaja, 2017).
- Treated cancer patients exposed to environmental stress leading to mitochondrial injury and mitochondrial DNA (mtDNA) extracellular release (Sansone et al., 2017). In both cases, 3PM approach is based on individualised patient profiling, predictive diagnostics and therapies tailored to the person. To this end, systemic effects are well reflected in the mitochondrial health status—the regular control of which is highly recommended for predictive and preventive purposes (Koklesova et al., 2022).

## Data Availability

The datasets presented in this study can be found in online repositories. The names of the repository/repositories and accession number(s) can be found in the article/supplementary material.
